# Synthetic Control Methodology for Examining Firearm Policy

**DOI:** 10.1007/s40471-022-00294-9

**Published:** 2022-07-19

**Authors:** Michelle Degli Esposti, Douglas Wiebe, Elinore Kaufman, Carl Bonander

**Affiliations:** 1 Penn Injury Science Center, University of Pennsylvania, Blockley Hall, 423 Guardian Dr, Room 902, Philadelphia, PA 19104-6021 USA; 2 Human Development and Violence Research Centre (DOVE), Federal University of Pelotas, Pelotas, Brazil; 3 Department of Social Policy and Intervention, University of Oxford, 32 Wellington Square, Oxford, UK; 4 Division of Traumatology, Surgical Critical Care, and Emergency Surgery, University of Pennsylvania Perelman School of Medicine, Philadelphia, USA; 5 Health Economics and Policy, School of Public Health and Community Medicine, Institute of Medicine, University of Gothenburg, Gothenburg, Sweden

**Keywords:** Synthetic control methodology, Evaluation methods, Firearm policy, Firearm law

## Abstract

**Purpose of Review:**

Firearm policies have the potential to alleviate the public health burden of firearm violence, yet it is unclear which policies are effective. The current review aims to summarize studies that use synthetic control methods to overcome previous methodological limitations when examining the impacts of firearm policies.

**Recent Findings:**

Evidence from studies using synthetic control methods find compelling evidence that purchasing licensing laws for all individuals (e.g., permit-to-purchase) have a preventive effect on firearm deaths. Otherwise, the effects of other firearm policies targeting firearm availability, ownership, sales, and use varied across studies and contexts.

**Summary:**

Synthetic control evaluations find heterogenous effects of firearm policies, suggesting that previous inconsistent findings might reflect their varying impacts across regions rather than methodological limitations alone. Future research should aim to exploit the complementary biases of synthetic control methods to triangulate evidence across evaluation approaches and understand why firearm policies have differential impacts.

## Introduction

Worldwide, more than 250,000 people die from firearm injuries each year [1]. These deaths are predominantly caused by lethal violence as homicide and suicide account for over 90% of firearm deaths. Firearm violence is increasingly recognized as a critical global public health problem, placing serious social and economic costs on societies in addition to the most serious cost of loss of life [1–3]. The burden of firearm mortality varies both between and within countries. Brazil and the United States (US) account for 32% of all firearm deaths globally [2], and rates are 12 times higher in the US compared to other high-income countries [4]. There is also substantial variation within the US, with increasing trends in some states (e.g., Missouri) and decreasing in others (e.g., California) [5]. Thus, firearm deaths are shaped by complex factors that differ by region, including policies and laws that governments use to regulate access to, and use of, firearms [6].

A growing number of studies examine the wide range of firearm policies that often aim to reduce avoidable firearm deaths (Table 1), particularly in the US. While there remains a lack of clarity on which policies effectively prevent firearm deaths [6–10] and which increase deaths [11], some consistent findings have emerged. First, the simultaneous adoption of multiple laws that target different elements of firearms regulation has led to reductions in firearm-related deaths in some countries [6], such as Australia [12, 13]. Second, more restrictive firearm policies are generally associated with decreased firearm deaths, as are stronger background checks and permit-to-purchase laws [6, 14]. Third, limitations in the evidence can be attributed in part to difficulties in providing valid estimates of the impacts of firearm policies [15].

**Table 1:** Categories of firearm policies

Target of the policy	Firearm policy	Description	Specific laws
Availability	Restrictions on firearms and/or ammunition	Laws prohibiting specific types of firearms, including automatic and semiautomatic firearms and poor-quality (junk) firearms; as well as laws banning specific types of ammunition (e.g., high-capacity ammunition magazines)	Assault weapons ban (AWB); Saturday Night Specials ban
	Gun buyback programs	Mandatory or voluntary buybacks of firearms, where firearms are collected from citizens and destroyed	Australia’s 1996 National Firearms Agreement (NFA)
Ownership	Purchasing licenses	Laws that determine whether a license or permit—issued by a government authority—is required for an individual to buy and own a firearm	Permit-to-purchase (PTP)
	Restrictions on individuals	Laws prohibiting the purchasing or possession (via seizures) of firearms for high-risk individuals, including felons, youths, and those with mental health conditions. Includes laws removing firearms where there is reasonable concern of violence	Minimum age; firearm seizure; misdemeanor violence prohibition (MVP)
	Firearm registration	Laws that require individuals to record their ownership of a firearm with a designated government agency (typically a law enforcement agency)	n/a
	Firearm safety training	Laws that require training on safe firearm to undergo some form of safety training prior to being able to purchase and/or carry a firearm	n/a
	Safe storage	Laws that require gun owners to store their firearms unloaded and locked when unattended in order to help prevent unauthorized users (e.g., children), from accessing and using firearms	Child access prevention (CAP)
	Reporting lost and stolen firearms	Laws that require firearm owners to notify law enforcement about the loss or theft of a firearm to deter gun trafficking, straw purchasing, and illegal possession	n/a
Sales	Dealer licenses and inspections	Laws that regulate firearm dealers, including requiring dealers to obtain a license and permit or require inspections of dealers	n/a
	Background checks	Laws that identify individuals who are ineligible to purchase firearms and prevent those persons from obtaining them. Background checks most commonly apply to sales from dealers but can also cover private sales	Comprehensive background checks (CBC)
	Record-keeping & reporting	Laws that specify record-keeping requirements where firearms dealers are required to collect and maintain sales records. In addition, reporting requirements require dealers to report specific events to a government agency, such as multiple firearm sales to the same purchaser within a certain time-period	n/a
	Waiting periods	Laws that prevent gun purchases from taking possession of their firearm immediately upon purchase and/or completion of a background check. Instead, these laws impose delays of days or weeks between the purchase and the date on which the buyer may take possession of the firearm	n/a
	Sales restrictions	Laws that determine specific conditions of firearm sales; both in terms of the quantity of sales and the type of firearm sold (see also Availability above)	One handgun a month; assault weapons ban (AWB); Saturday Night Specials ban
	Gun shows	Laws regulating gun shows, including zoning ordinances barring gun shows on public property	n/a
Use	Firearm carry	Laws that allow individuals to carry firearms in public places. Includes laws specifying the carrying of concealed weapons (CCW), as well as open carry where the firearm is visible	Shall-issue right to carry (RTC); may-issue; open carry
	Self-defense	Laws that determine individual rights on the use of lethal violence, including the use of firearms, in self-defense; from public places to an individual’s property (home, vehicle, workplace)	Stand your ground (SYG); castle doctrine
	Location restrictions	Laws that prohibit the possession of firearms in specific locations, such as airplanes, post offices, government buildings, and public schools/colleagues/universities	Gun-free zones; gun-free school zones act (GFSZA)
	Firearm misuse	Laws that punish the misuse of firearms, including publicly firing a gun or in other banned locations (e.g., private shooting ranges)	n/a
	Hunting restrictions	Laws specifying restrictions on the use of firearms for hunting	n/a

Adapted from Cook and Goss [28], Santaella-Tenorio et al. [6], and the Giffords Law Center and RAND online resources [29, 30]

n/a, Not applicable

Firearm laws are heterogeneous in formulation, implementation, and enforcement [16]. Comparing firearm laws across regions and periods can be difficult because law changes are not always immediately implemented or enforced, and public response may be delayed [8]. Other challenges include difficulties in identifying appropriate control groups and fulfilling the modeling assumptions underlying the common analytical techniques used to evaluate firearm policies, such as interrupted time series (ITS) and difference-in-difference (DiD) designs [17, 18]. Moreover, a recent simulation study showed that the existing literature suffers from sensitivity to modeling specifications and commonly used modeling approaches in gun policy research have high rates of false positives [15, 19]. Subsequent recommendations have called for future research to use alternative evaluation approaches to avoid further hindrance from the limited power of traditional significance testing methods [15].

Synthetic control methodology (SCM) offers an alternative to traditional evaluation methods and can overcome key limitations in the firearm policy literature: (i) the lack of comparable intervention and control groups, and (ii) fragility of findings to modeling specifications. SCM is a data-driven technique for evaluating the impacts of population-level interventions [20–23], such as firearm policies, and has been described as “the most important innovation in the evaluation literature in the last fifteen years” [24]. The method moves away from traditional significance testing methods by using a data-driven algorithm to identify an optimal weighted control unit—a “synthetic control”—based on pre-intervention data from available control units, referred to as the “donor pool” [20–22]. The method aims to construct a well-matched counterfactual for between-group studies by minimizing systematic differences between the intervention and control units’ pre-intervention outcome trends (and covariates) [25]. The approach uses optimization to determine the weights for each potential control and variable importance from the covariates to construct the synthetic control unit. Any post-intervention differences between the intervention and synthetic control unit should be attributable to the intervention itself, provided the synthetic control matches the intervention unit on a long series of pre-intervention outcomes, and no other firearm policies are implemented during the study period [21]. SCM neither relies on traditional significance testing nor requires strict modeling assumptions about the shape of the intervention effect over time, and thus addresses some of the current limitations in the firearm policy literature. The method was initially developed for interventions implemented in a single unit (e.g., the introduction of a national firearm law) [20, 21] but has since been generalized to more complex data structures (e.g., multiple intervention units with staggered adoption) [26, 27]. Given the potential and relative infancy of the method, this review aims to summarize and critically evaluate the evidence SCM has generated on firearm policy effects.

## Methods

We systematically searched the literature from January 2015 to November 2021, when the searches were performed. We searched for published studies in 5 databases: Embase, Pubmed, Proquest, PsycINFO, and Ovid Medline. Following a scoping search for empirical studies that used synthetic control methodology to examine firearm policies, we searched for articles that contained the following terms in the title and/or abstract: (“gun” or “guns” or handgun* or firearm*) AND (“legislation” or “law” or “laws” or “statute” or “statutes” or “regulation” or “policy” or “jurisprudence”) AND synthetic control*. We also conducted directed searches of Google Scholar and the RAND Corporation, examined bibliographies of relevant articles, and included papers previously known to the authors.

We identified 88 articles from databases, 1 article from the RAND Corporation [31], and 4 articles previously known to the authors [23, 32–33]. Articles were de-duplicated, screened, and evaluated for relevance. For this narrative review, we excluded 3 theses and dissertations [34–36]. This resulted in 17 empirical studies and two commentaries on the included empirical papers [37, 38]. Four methodological papers were also identified but excluded [23, 31, 32, 33].

## Methodological Observations

Included studies that used synthetic control methodology (SCM) to examine firearm policies are summarized in Table 2. Among the 17 studies, only one was conducted outside of the US (in Australia) [39••]. This was also the only study to evaluate a national firearm policy [39••]. All 17 studies applied the original single-intervention-unit SCM [20, 21], irrespective of the number of intervention units (range 1–33). Studies with more than one intervention unit were exclusively evaluations of firearm policies across US states. The most common inference method was the in-place placebo test [21] (used in 16 of the 17 studies). Four studies also used unverified inference methods, including conventional *t*-tests to test for differences in the treated and synthetic control outcomes [40•, 41, 42] and segmented regression to identify structural breaks in the synthetic control outcomes [32]. Only a handful of academics published papers using SCM to examine firearm policies, often authoring more than one included study. For example, some authors appeared in a quarter of all studies identified and one academic co-authored seven included studies. This is likely due to SCM not yet being widely understood and applied in public health research [23, 25], and thus only a handful of academics are well versed in both methodology and topic.

**Table 2:** Summary of studies examining firearm policies using synthetic control methodology

Reference	Intervention		Study design					Analysis		Findings	
	Firearm policy, county	Unit (date)	Evaluation method	Donor pool(s)	Study period (interval)	Outcome, source	Inclusion of covariates	SCM inference method	Other analyses	SCM results	Robust to analytical approach?
Bartos et al. [39••]	1996 Gun Buyback Program, Australia	Australia (1997)	Single-unit SCM	28 WHO nations with similar population sizes & minimal missing data	1967–2007 (yearly)	Homicide & suicide, WHO	Did not match on covariates due to unbalanced (missing) data and because the causal process of homicide is unknown	Placebo-tests (in-place)	Negative control (motor vehicle fatalities)	Australia’s 1996 Gun Buyback Program led to significant reductions in homicide rates but not suicide rates	Yes
Bhatt et al. [49•]	Permit-To-Purchase [repeal] and Carrying a Concealed Weapon [lowered age limit], US	Missouri (PTP repeal: 2007; CCW: 2011 & 2014)	Single-unit SCM	States without related policies during study period (13 states for PTP; 42 states for CCW)	1999–2018 (yearly)	Firearm & non-firearm suicide for adolescents & young adults, Vital Statistics CDC	Matched on covariates that are hypothesized/evidenced predictors of suicide	Placebo-tests (in-place)	No	The repeal of Missouri’s PTP was associated with a 22% increase in firearm suicide rates among 19- to 24-year olds The lowering of the minimum age of CCW laws in Missouri was associated with a 32% increase in firearm suicide rates among 14- to 18-year olds and 7% increase among 19- to 24-year olds	n/a
Castillo-Carniglia et al. [44]	Comprehensive Background Checks, US	Delaware (July 2013); Colorado (July 2013); Washington (Dec 2014)	Single-unit SCM	29 states without related policies during study period	Jan 1999–Dec 2016 (monthly)	Background checks, FBI NICBCS	Matched on covariates that are hypothesized/evidenced predictors of background checks (including a systematic literature search)	Placebo-tests (in-place)	Sensitivity analysis of model specifications (predictor & outcome inclusion) ITS analysis	The enactment of CBC laws was associated with an increase in background checks in only Delaware. No effect was seen in Colorado and Washington	Moderate – SCM results robust to model specifications but modest deviation between SCM and ITS analysis as effects were only significant in Delaware at the mid-post implementation period for the ITS analysis
Castillo-Carniglia et al. [43•]	Comprehensive Background Checks and Misdemeanour Violence Prohibition, US	California (1991)	Single-unit SCM	32 states without related policies during study period	1981–2000 (yearly)	Firearm homicide & suicide, Vital Statistics CDC	Matched on covariates that are hypothesized/evidenced predictors of homicide & suicide, and retained covariates that showed preferential model performance (lowest RMSPE)	Placebo-tests (in-place)	Sensitivity analysis of model specifications (donor pool restriction) Negative control (non-firearm homicide & suicide)	California’s CBC and MVP policies were not associated with significant changes in firearm suicide or homicide rates	Yes
Castillo-Carniglia et al. [48]	Comprehensive Background Checks, US	Washington (Dec 2014); Oregon (Aug 2015)	Single-unit SCM	28 states without related policies during study period	Jan 1999–Dec 2018 (monthly)	Background checks, FBI NICBCS	Matched on covariates that are hypothesized/evidenced predictors of background checks	Placebo-tests (in-place)	No	Oregon’s CBC law was associated with an increase in background checks, but no significant increase was seen for Washington’s CBC law	n/a
Crifasi et al. [45]	Permit-To-Purchase [enactment & repeal], US	Connecticut (enactment, 1995) & Missouri (repeal, 2007)	Single-unit SCM	States without related policies during study period: 39 for Connecticut and 48 for Missouri	1981–2012 (yearly)	Firearm & non-firearm suicide, WISQARS	Matched on covariates that are hypothesized/evidenced predictors of suicide	Placebo-tests (in-place)	ITS analysis	Connecticut’s PTP law enactment was associated with a 15% reduction in firearm suicide rates; Missouri’s PTP law repeal was associated with a 16% increase in firearm suicide rates	Moderate – ITS analysis identified a similar reduction following Connecticut’s PTP law but did not replicate findings in Missouri
Rudolph et al. [50]	Permit-To-Purchase, US	Connecticut (1995)	Single-unit SCM	39 states without related policies during study period	1984–2005 (yearly)	Firearm & non-firearm homicide, WISQARS	Matched on covariates but did not specify inclusion criteria	Placebo-tests (in-place)	Non-weighted DiD analysis	Connecticut’s PTP law was associated with a 40% reduction in firearm homicide rates	Yes –DiD analysis also identified a reduction in firearm homicide, albeit smaller than that identified by SCM
Gius [41]	Firearm Seizure, US	Connecticut (1999); Indiana (2005)	Single-unit SCM	33 states without related policies during study period & no missing data	1990–2017 (yearly)	Homicide & firearm homicide, SHR US Department of Justice	Matched on covariates that were used in previous research	Placebo-tests (in-place) *t*-tests for significant differences pre- & post-intervention period	No	Firearm seizure laws were associated with a reduction in homicide and firearm homicide in Connecticut but an increase in firearm homicide in Indiana	n/a
Gius [40•]	Child Access Prevention, US	22 states^*^ (range: 1990–2010)	Single-unit SCM	23 states^†^ without related policies during study period	1981–2017 (yearly)	Youth suicide, WISQARS	Matched on covariates that were used in previous research	Placebo-tests (in-place) *t*-tests for significant differences pre- & post-intervention period. *t*-tests that were significant in the pre-intervention period were excluded	No	CAP laws were associated with reductions in youth firearm suicide rates in 9 states but showed no significant effects in 13 states	n/a
Gius [42]	Right-To-Carry, US	8 states^*^ (range: 1995–2006)	Single-unit SCM	States^‡^ without related policies during study period & no missing data	1990–2014 (yearly)	Homicide & firearm homicide, SHR US Department of Justice	Matched on covariates that were used in previous research	Placebo-tests (in-place) *t*-tests for significant differences pre- & post-intervention period. *t*-tests that were significant in the pre-intervention period were excluded	Non-weighted DiD analysis	Limited evidence that making CCW laws more permissive—moving from prohibited to shall issue RTC status—impacted homicide or firearm homicide. Only New Mexico was associated with an increase in homicide or firearm homicide rates, there was no significant change in the seven remaining states	Moderate – DiD analysis (fixed effects model) identified an overall significant increase in homicide and firearm homicide
Guettabi et al. [58••]	Stand Your Ground, US	14 states^*^ (range: 2005–2007)	Single-unit SCM	20 states without related policies during study period	Deaths: 1991–2011 (yearly) Firearm homicide: 1991–2012 (yearly)	Firearm death (excluding suicide) & homicide, Vital Statistics CDC & FBI UCR	Matched on covariates that were used in previous research	Placebo-tests (in-place)	Non-weighted DiD analysis Negative control (firearm suicide)	SYG laws were associated with increases in firearm death rates in three (Florida, Alabama, Michigan) out of 14 states and increases in homicide rates in only Florida	Yes
Kagawa et al. [46•]	Comprehensive Background Checks [repeal], US	Indiana & Tennessee (1998)	Single-unit SCM	9 states with related policies during study period and without sparse data	Indiana: 1981–2008 (yearly) Tennessee: 1994–2008 (yearly)	Firearm & non-firearm deaths (homicide & suicide), WISQARS	Matched on covariates but did not specify inclusion criteria	Placebo-tests (in-place)	Non-weighted DiD analysis	The repeal of CBC laws was not associated with a change in firearm homicide or firearm suicide rates in Indiana and Tennessee	Yes
Kahane et al. [52•]	Gun Law Reform, US	Massachusetts (1998)	Single-unit SCM	49 states without the same gun law reform	1981–2007 (yearly)	Suicide & firearm suicide, Vital Statistics CDC	Matched on covariates but did not specify inclusion criteria	Placebo-tests (in-place)	No	Changes to 23 gun laws in Massachusetts that primarily placed restrictions on firearm ownership was associated with an initial reduction in total suicide rates and a sustained reduction in firearm suicide rates	n/a
Kivisto et al. [47•]	Firearm Seizure, US	Connecticut (1999); Indiana (2005)	Single-unit SCM	States without related policies during the study period: 48 for Connecticut & 47 for Indiana	1981–2015 (yearly)	Suicide (firearm & non-firearm suicide), WISQARS	Matched on covariates that are hypothesized/evidenced predictors of suicide	Placebo-tests (in-place)	Non-weighted DiD analysis	Connecticut’s firearm seizure law was initially associated with a 2% reduction in firearm suicide rates, which then increased to a 14% reduction in the period after the Virginia Tech mass shooting Indiana’s firearm seizure law was associated with an 8% reduction in firearm suicide rates	Yes
McCourt et al. [51••]	Permit-To-Purchase [enactment & repeal] and Comprehensive Background Checks [enactment], US	Connecticut (PTP enactment, 1995); Missouri (PTP repeal, 2007); Pennsylvania (CBC enactment, 1995); Maryland (CBC enactment, 1996)	Single-unit SCM	29–39 states without related policies during the study period	1985–2017 (yearly)	Firearm & non-firearm homicide & suicide, Vital Statistics CDC	Matched on covariates that are hypothesized/evidenced predictors of homicide & suicide	Placebo-tests (in-place)	No	Connecticut’s PTP law enactment was associated with a 28% reduction in firearm homicide rates and a 33% reduction in firearm suicide rates; Missouri’s PTP repeal was associated with a 47% increase in firearm homicide rates and a 24% increase in firearm suicide rates There was no clear evidence of an association between Maryland’s or Pennsylvania’s enactment of CBC laws on firearm mortality rates.	n/a
Degli Esposti et al. [59]	Stand Your Ground, US	Florida (Oct 2005)	Single-unit SCM	16 states without related policies during the study period	January 1999 and December 2017 (quarterly)	Firearm homicide for adolescents, Vital Statistics CDC	Matched on covariates that are hypothesized/evidenced predictors of homicide	ITS analysis as SCM was a sensitivity analysis only	ITS analysis Negative control (firearm suicide)	Florida’s SYG law associated was with an increase in firearm homicide among adolescents	Yes
Donohue et al. [57••]	Right-To-Carry, US	33 states^*^ (range: 1981–2007)	Single-unit SCM	28 states without related policies during the study period	1977–2014 (yearly)	Violent & property crime, and homicide, FBI UCR & Vital Statistics CDC	Matched on covariates that are hypothesized/evidenced predictors of crime & violence	Placebo-tests (in-place) Tests of influential control units in donor pool	Non-weighted DiD analysis	RTC concealed guns laws were associated with a 13%–15% increase in violent crime rates 10 years after the laws were introduced. No significant effect was found for property crime or homicide rates	Yes

Abbreviations: CAP, Child access prevention; CBC, Comprehensive background checks; CCW, Carrying a concealed weapon; CDC, Centers for disease control and prevention; DiD, Difference-in-differences; FBI, Federal Bureau of Investigation; ITS, Interrupted time series; MVP, Misdemeanor violence prohibition; n/a, Not applicable; NICBS, National Instant Criminal Background Check System; PTP, Permit-to-purchase; RTC, Right-to-carry; SHR, Supplementary Homicide Reports: RMSPE, root mean squared prediction error; SYG, Stand your ground; UCR, Uniform crime reporting; US, United states; WHO, World Health Organization; WISQARS, Web-based Injury Statistics Query and Reporting System

^*^Studies examining more than five intervention units were summarized rather than providing details for each unit.

^†^Number of states in donor pool deduced from all states (*n* = 50) minus treatment pool (*n* = 27). Number of control units in the donor pool is not clearly specified in the original manuscript

^‡^Number of control units in the donor pool is unclear in the original manuscript

SCM was generally applied with methodological rigor. Evaluations were generally sensitive to contamination effects and appropriately excluded controls that may have been influenced by the intervention from the donor pool. For example, all 16 US studies excluded states (i.e., controls) from the donor pool that had similar policies enacted, or not enacted if evaluating a policy repeal, during the study period (Table 2). The only non-US study evaluating Australia’s 1996 Gun Buyback program did not specifically exclude other countries (i.e., controls) with gun buyback programs but did restrict the donor pool to countries with population sizes greater than 500,000 [39••]. Four additional studies excluded controls that had sparse and/or missing data [39••, 41, 46, 49•]. A few studies conducted sensitivity analyses to check the robustness of findings to donor pool restrictions [43•, 44].

All but one study matched on and adjusted for a comprehensive list of covariates in the synthetic control models (Table 2). Most covariates were selected and included as they were state-level characteristics hypothesized and/or evidenced to predict the outcome(s) under evaluation, namely homicide and suicide. Common covariates thus included measures of population sociodemographics (age, sex, race, ethnic distribution), geography (population density, urbanicity, metropolitan statistical areas), and economics (e.g., poverty, unemployment rate, median household income, Gini coefficient, high school education), as well as alcohol consumption and proxy measures of gun availability. Less common covariates included rate of religious adherence [51••], number of law enforcement employees [51••], and violent crime rates [44, 46, 47•, 48]. One study selected covariates which further improved synthetic control model fit [43•], and three studies did not fully explain why covariates were included [46, 50, 52•]. The only study to not directly adjust for covariates was the national evaluation of Australia’s 1996 Gun Buyback program, which described common covariates but did not include them in the synthetic control models due to unbalanced (missing) data among control countries in the donor pool [39••]. The authors’ further argued that, because the causal process of homicide is unknown, it is preferential to match on the pre-intervention trend in the outcome series rather than covariates [39••].

Despite this, there was often poor information on included matching variables, including if they were time constant or varying, their time intervals and period, and their variable importance weights (determined by the optimization). Several studies did not achieve a good match on pre-intervention trends, which we identified to be mainly due to volatile and/or sparse outcomes. Although most studies tried to minimize related biases by including robustness checks or alternative evaluation methods [43•, 44, 49•], other studies did not comprehensively address poor synthetic control fit [40•].

## Empirical Evidence

### Policies Targeting Availability

We identified only one study that used SCM to evaluate policies that target firearm availability. The study examined the nationwide rollout of Australia’s Gun Buyback Program, which ran for 12 months from October 1996 to September 1997 and was estimated to retrieve around 650,000 guns [39••]. Australia was compared to a weighted control unit derived from 28 WHO-reporting countries with similar population sizes and minimal missing data. The study found significant reductions in homicide rates and no effect on their negative control outcome of motor vehicle fatalities following the 1996 gun buyback program [39••]. However, there was also no significant effect on suicide rates. The authors argued that this absence of effect was due to the 1996 program being limited to military-style assault rifles and shotguns.

### Policies Targeting Ownership

**Fig. 1:**
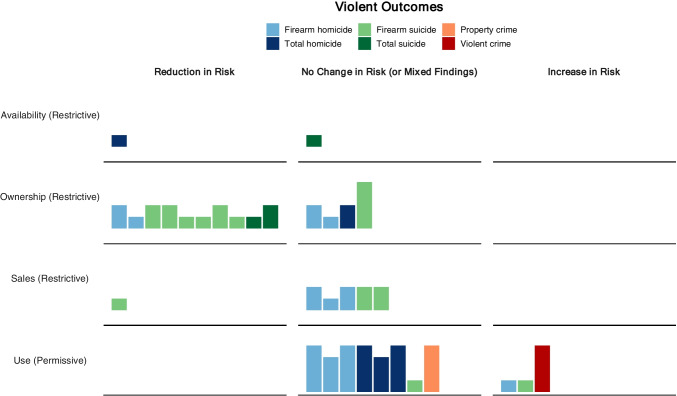
Harvest plot on the direction of effect across category of firearm policy, type of violent outcome, and number of intervention units (bar height: 1; 2–5; 6–10; 10 +)

There were several evaluations of different policies that targeted firearm ownership. This included four studies evaluating purchasing licenses (permit-to-purchase laws) [45, 49•, 50, 51••], three studies evaluating restrictions on individuals (firearm seizures and misdemeanor violence prohibition laws) [41, 43•, 47•], and one study evaluating safe storage (child access prevention laws) [40•]. An additional study evaluated Massachusetts Gun Law Reform in 1998, which made unprecedented changes to state firearm laws through 23 legislative changes [52•]. These primarily placed restrictions on gun ownership through individual restrictions (minimum age, misdemeanor violence prohibition), firearm safety training, safety storage, and reporting lost and stolen firearms requirements. This evaluation found a reduction in total firearm suicide rates for several years and a sustained reduction in firearm suicide rates after placing additional restrictions on gun ownership [52•]. Overall, there was consistent evidence of a preventive effect of purchasing licenses on firearm mortalities but inconsistent evidence on the effectiveness of restricting ownership for specific individuals and safe storage requirements (see Fig. 1).

#### Purchasing Licenses

Permit-to-purchase (PTP) law requires a prospective firearm buyer to apply for a license directly to a government authority (e.g., local law enforcement agency) that vets the application and initiates a background check. Four studies used SCM to evaluate the impacts of PTP laws on firearm suicide and homicide rates. Studies consistently found reductions in firearm suicide and homicide rates following the introduction of PTP laws and increases after the law was repealed [45, 49•, 50, 51••]. The introduction of PTP laws was associated with a 28–40% decrease in firearm homicides and a 15–28% decrease in firearm suicides, while repealing PTP laws was associated with a 47% increase in homicides and 16–24% increase in suicides [45, 49•, 50, 51••]. In addition, two studies analyzed the repeal of Missouri’s PTP law on firearm suicide rates among adolescents and young adults specifically and reported similar increases among this age group [45, 49•]. This literature is limited by only examining the impact of PTP laws in two states: the enactment of Connecticut’s PTP law in 1995 [45, 50, 51••] and the repeal of Missouri’s PTP law in 2007 [45, 49•, 51••].

#### Restrictions on Individuals

There was mixed evidence on the effectiveness of firearm seizure laws by state, though evidence trended towards reductions in firearm deaths. In 1999, Connecticut became the first state to enact firearm seizure legislation following a mass shooting at the state lottery headquarters. Two examinations of Connecticut’s firearm seizure law found associated reductions in firearm suicide and homicide rates [41, 47•]. In addition, the reduction in firearm suicide rates was more pronounced following the Virginia Tech mass shooting, which led to a fivefold increase in the number of guns seized [47•]. Indiana’s firearm seizure law, enacted in 2005, was also associated with a decrease in firearm suicide rates [47•]. However, a second study identified an increase in firearm homicide rates following Indiana’s firearm seizure law [41]. An additional examination of California’s enactment of a misdemeanor violence prohibition (MVP) policy, alongside the enactment of a comprehensive background check, found no change in either firearm homicides or suicides [43•]. The reason for varying effects across states is unclear but may be linked to differences in the laws themselves or their implementation. For example, Connecticut’s law authorizes the seizure of firearms from individuals deemed a threat to themselves or others, whereas Indiana’s law requires the individual to both be a threat *and* mentally ill or violently unstable. Such legislative differences may be vital in determining whether firearm seizure laws effectively prevent firearm deaths, especially homicides.

#### Safe Storage

We identified one SCM evaluation of the safe storage requirement of child access prevention (CAP) laws across 22 US states on youth firearm suicide rates [40•]. The findings were mixed, with CAP laws being associated with lower youth firearm suicide rates in only nine out of 22 states. These results suggest that for some (but not all) US states, there was a preventive effect of CAP laws on firearm suicides among 0- to 18-year olds. However, these findings are subject to potential methodological limitations due to sparse and volatile data (see “Discussion” section).

### Policies Targeting Sales

We identified five studies that applied SCM to examine policies targeting firearm sales, specifically the impacts of comprehensive background checks (CBC) [43•, 44, 46, 48, 51••]. There were heterogeneous effects of CBC laws across states for background checks and null effects for firearm deaths. Delaware and Oregon’s CBC laws were associated with increases in the number of backgrounds checks, but no effect was seen in Washington and Colorado [44, 48], even though the law was implemented during a similar period (2013–2015). The authors suggested that the discrepancy in effects may be due to low compliance and/or insufficient enforcement of the laws in the states will null findings. When Washington’s CBC law was enacted (2014), there was a well-documented “I will not comply” rally in the state capital [53], while many county law enforcement officials in Colorado reported that they would not enforce its CBC law [54]. McCourt et al. [51••] found that the CBC laws in Maryland and Pennsylvania were not associated with reductions in firearm deaths. Castillo-Carniglia et al. [43•] reached the same conclusion about Calfornia’s CBC law. A further study found that the repeal of CBC laws in Indiana and Tennessee did not appear to reduce firearm suicides or homicides [46]. All three studies cite widespread non-compliance and infrequent enforcement of CBC laws in these states as a plausible explanation to why CBC laws are not consistently associated with reductions in firearm mortality rates [55, 56]. Together, the evidence from SCM studies suggests that CBC laws alone (i.e., in the absence of PTP laws), and when poorly enforced and complied with, may be insufficient for preventing firearm deaths.

### Policies Targeting Use

Two policies that targeted firearm use were evaluated using SCM. Three studies examined the impact of concealed carry laws [42, 49•, 57••], while two studies examined the impact of expanding an individual’s right to use firearms in self-defense [58••, 59]. The enactment of these policies made state firearm legalization more permissive. The estimated effects of both policies were heterogeneous across states. Stand your ground (SYG) laws were associated with robust increases in firearm homicide in some states (e.g., Florida) but not others (e.g., Indiana), and there was inconsistent evidence on whether relaxing concealed carry laws resulted in higher violence rates.

#### Concealed Carry

A comprehensive US-wide analysis of 33 states found that the enactment of “shall issue” right-to-carry (RTC) laws was associated with an increase in violent crime rates [57••]. But the authors found no robust association between enacting RTC laws and homicide and property crime. A subsequent commentary was published in response to this study and questioned the robustness of these findings [38]. The commentary replicated their analysis and found that it was more common to see reductions rather than increases in violent crime after enacting RTC laws. Another US-wide analysis also found limited evidence of the impact of RTC laws on homicide, with only one (New Mexico) out of eight states showing an increase in homicide and firearm homicide rates following the move from prohibited to RTC status [42]. A single-state analysis of Missouri, however, did report a 32% increase in youth firearm suicides when the minimum legal age to obtain a concealed carry permit was lowered to 19 years [49•]. A similar increase was seen for non-firearm suicide, questioning whether the observed effect resulted from this legal change.

#### Self-defense

Two studies examined the impact of expanding an individual’s right to use guns in self-defense by enacting SYG laws. A US-wide examination of 14 states reported heterogeneous effects of SYG across states, which varied in magnitude [58••]. Three (Florida, Alabama, Michigan) out of 14 states showed increases in firearm death rates (excluding suicide) that ranged from 13 to 24%. Florida had the largest increase in firearm deaths of 24% and was the only state to show an increase in homicide rates (of 13%). Moreover, an examination of Florida alone reported increases in firearm homicides among adolescents in Florida following the enactment of its SYG law, especially among African American adolescents [59].

## Discussion

### Methodological Value for Firearm Research

Robust evaluation designs are particularly important for firearm research given the controversy surrounding firearm policy and limitations in the current evidence base due to methodological inconsistencies [60]. Our review suggests that SCM has added methodological value to the firearm literature. First, SCM was used as an alternative method to complement more traditional evaluation approaches, such as DiD and ITS designs. For instance, nine of the 17 included studies used SCM to evaluate firearm policies alongside other analytical approaches and SCM broadly agreed with these other approaches [42, 43•, 45, 46, 47•, 50, 57••, 58••, 59]. Thus, SCM often served as a tool to assess the robustness of findings to different modeling approaches and replicate previous or concurrent examinations of firearm policies.

Second, SCM has been used to examine firearm policies in sparse data contexts where other evaluations would have been underpowered. Because SCM does not rely on formal frequentist inference, it has less stringent data requirements than other evaluation approaches like ITS analyses [23, 61]. A rigorous ITS analysis may require 20–30 pre-intervention data points to model underlying trends and seasonality [17]. SCM does not necessarily need as many pre-intervention data points (yearly data are usually sufficient). Most included studies (*n* = 14) relied on yearly outcome data, which would typically lead to poor statistical power in ITS evaluations.

Third, SCM was used to evaluate more complex and/or combined interventions, which often changed during the period after the first intervention was implemented. For example, Bhatt et al. [49•] evaluated the impacts of introducing three changes to firearm laws in Missouri: the repeal of PTP laws in 2007, and the lowering of the legal age to obtain a CCW from 23 to 21 years in 2010 and then to 21 to 19 years in 2014. The staggered introduction of multiple interventions in one state (i.e., same intervention unit) would be challenging to analyze using the more traditional method of ITS given the need for an impact model specifying the shape of the intervention effect over time, making it difficult to evaluate multiple interventions that rolled out gradually. SCM, however, offers a more flexible approach for evaluating these “messier” interventions since time-varying impacts can be estimated without specifying an impact model. The impact of firearm laws can therefore be assessed without fragile modeling assumptions [23].

### Summary of the Empirical Evidence

The examinations of firearm policies using SCM primarily contributed to the literature by bolstering existing findings. The studies echo much of the inconsistent evidence in firearm research and the heterogeneous effects of firearm policies (see Fig. 1). This suggests that inconsistencies in the current evidence base are not solely due to modeling misspecifications [6, 60]—as SCM counteracts many previous concerns—but may also reflect the varying nature of the impacts of firearm policies themselves. Firearm policies are rarely homogenously formulated, implemented, and enforced across contexts [16]. In addition, changes to firearm policies do not occur in a vacuum but against a backdrop of several types of existing laws and contributing factors, such as the built environment, economic trends, population characteristics, media attention, culture, and law enforcement. Firearm policies most likely interact with these pre-existing laws and characteristics to shape heterogeneous effects across contexts. While it is difficult to tease apart whether inconsistent evidence is due to fragility to modeling approaches or effect heterogeneity, SCM has helped strengthen the latter interpretation for specific firearm policies. For example, SYG self-defense laws have been found to increase homicides in some states (e.g., Florida) but not others (e.g., Indiana) across evaluation designs: SCM [58••], ITS [62], and DiD [11]. This suggests that the variation is not simply caused by methodological issues related to specific methods and may instead capture effect heterogeneity across regions (and time) [63].

Although the SCM evidence indicated heterogeneous effects of firearm policies, there was compelling evidence that purchasing license (PTP) laws have a preventive impact on firearm homicides and suicides (Fig. 1). Reductions in firearm deaths have been observed after the introduction of PTP laws, and increases have been observed when they are repealed. These findings replicate existing literature [6], suggesting that laws that regulate firearm purchases and ownership for all individuals effectively prevent firearm deaths. Our review identified few controversial findings. A SCM analysis of 22 US states found mixed evidence of a preventative effect of CAP laws on youth suicide, with most states showing null effects [40•]. This finding contradicts previous literature, and the author argues that CAP laws may be less effective at reducing youth firearm suicides than prior research suggests [64–66]. However, these findings were based on synthetic controls with questionable validity due to sparse and volatile outcome data (e.g., Rhode Island). The null findings identified by this study may therefore represent inadequate synthetic control fit rather than true null effects of CAP laws in these states.

## Recommendations

To exploit the potential of synthetic control methodology in firearm research fully, we make several recommendations. First, SCM should be more widely applied to examine firearm policies beyond the US context. Second, the added value of SCM for examining firearm research pivots around the method being usable with small datasets and not requiring strong modeling assumptions. SCM can thus address biases in alternative evaluation methods, including DiD and ITS [19]. In addition, SCM can identify time-varying effects and can therefore be used to study policy changes that are gradually implemented over time.

Third, future studies should exploit the increasing number of generalizations that have been formulated in recent years. Notably, SCM has now been extended for evaluating multiple and staggered interventions (e.g., generalized and augmented synthetic control methodologies [26, 27]), which would have been informative analytical tools for several of the US-wide studies identified in this review [40•, 42, 57••, 58••]. Among these included studies, Gius [40•] examined the staggered adoption of CAP laws in 22 states, Guettabi [58••] of SYG laws in 14 states, and Donohue [57••] of RTC laws in 33 states across the US. These studies applied single-unit SCM, separately running SCM for each state that adopted the laws and then averaging these estimates [27, 57••]. This approach is computationally unwieldy and not well understood [27]. It potentially incurs bias due to overlapping donor pools as control units often appear in more than one synthetic control, and there is a lack of clarity on how to handle treated units with poor synthetic control fit [27]. Gius [40•] excluded states that showed poor synthetic control fit to address this issue but it is unclear if this introduces its own selection bias. Both generalized and augmented SCM offer the opportunity to evaluate similar firearm policies adopted at the same and/or different times across different units, such as states [26, 27]. These extensions simultaneously estimate an intervention effect at the unit (e.g., state) and aggregate level (e.g., national). Additionally, these extensions strengthen causal inference and overcome inference issues of single-unit SCM as the methods provide uncertainty estimates that conform to more conventional statistical inference, such as confidence intervals using the parametric bootstrap procedure [26].

Finally, as synthetic control methods gain traction and popularity, we caution against SCM being inappropriately and inconsistently applied and underline the importance of standardized reporting. While SCM was generally used with methodological rigor, a handful of studies poorly specified which variables (and their temporal information) were used in the matching process, did not adequately address synthetic control fit, and used unverified methods for statistical inference [40•, 41, 42]. Future research that encounters poor pre-intervention fit due to sparse outcome data should consider matching on smoothed data or using recently proposed bias-correction methods [27, 67, 68]. In addition, future studies should avoid using conventional *t*-tests to test for differences between the intervention and synthetic control unit because it does not account for the construction of the synthetic control weights [69, 70]. To guard against potential biases, we emphasize the importance of standardizing the application of the method and identified a unified approach to statistical inference, such as placebo tests [21] or new inferential methods developed for SCM [69–73]. We point future firearm research towards recent tutorials that guide researchers through key assumptions and requirements of synthetic control evaluations [23, 61], and provide an overview of key limitations to consider below.

### Limitations of SCM for Firearm Research

SCM is an increasingly flexible method that can be used to evaluate firearm policies implemented in discrete areas, such as state or national firearm legislation [23, 61]. Nevertheless, there are key limitations that need to be considered before applying SCM. Similarly to most controlled before-after study designs, evaluators should determine whether the following biases are potent: (1) anticipation bias where the treated area responds to the firearm policy before it is officially enacted; (2) contamination/spillover effects of the firearm policy to control units (e.g., neighboring areas); (3) co-interventions that occur at the same time as the firearm policy (e.g., a recording change to the outcome data); and (4) time-varying confounders in the post-intervention period that do not equivalently affect the treated and synthetic control unit (e.g., COVID-19 pandemic disproportionally impacting the treated area) [32].

Additionally, there are a number of practical and methodological issues to consider when determining the appropriateness of SCM [23]. The more common issues for firearm research include the problem of the convex hull, sparse and noisy outcome data, and poor synthetic control fit (including overfitting). While some of these issues can be overcome [23], the convex hull is an essential condition of SCM which requires the pre-intervention data of the treated unit to fall within the range of the donor pool. This condition cannot always be met. For example, an evaluation of a national firearm policy on gun deaths in Brazil may not be possible as firearm death rates typically exceed other national rates around the world. Sparse and missing outcome data may also present obstacles when examining the impact of policies on infrequent events, such as unintentional firearm deaths or child firearm suicide, as such data may prevent the identification of a good fitting synthetic control to the underlying trends.

## Conclusions

Synthetic control methods can meaningfully contribute to the field by offering a complementary evaluation approach for examining firearm policies. To date, SCM has primarily been used to replicate existing findings and the evidence generated from SCM studies continue to show heterogenous effects of firearm policies. This suggests that the current evidence is not purely inconsistent due to methodological limitations in conventional approaches. Although methodological limitations and differences across analytical approaches continue to contribute to observed effect heterogeneity, the SCM evidence reviewed here indicates that inconsistent findings may also reflect true heterogenous effects of certain firearm policies across different contexts. To advance the field further, future research should aim to explicitly exploit the data-driven algorithms and non-frequentist approach of SCM (and its extensions) to triangulate evidence across evaluation approaches with complementary biases [63]. Only then, can evaluations of firearm policies obtain more reliable answers and move closer towards understanding if, how, and why firearm policies have differential impacts.
